# Interaction of a genetic sum score of risk alleles associated with coronary artery disease by physical activity in the Heinz Nixdorf Recall study

**DOI:** 10.1186/s12872-026-06340-4

**Published:** 2026-07-25

**Authors:** Johannes Penther, Nicola Wilkening, Laven Mavarani, Raimund Erbel, Börge Schmidt

**Affiliations:** Institute for Medical Informatics, Biometry and Epidemiology, University Hospital Essen, Hufelandstr. 55, Essen, 45122 Germany

## Abstract

**Objectives:**

This study aimed to investigate the interaction between a genetic risk score for coronary artery disease (CAD) and measures of physical activity on coronary artery calcification (CAC) in a population-based cohort. Specifically, it sought to determine whether physical exercise, known to be protective against high CAC, influences the expression of genetic risk factors for CAD.

**Methods:**

Data were obtained from the Heinz Nixdorf Recall study, including 3938 participants aged 45–74 years with European ancestry. CAC was measured using electron beam computed tomography. The genetic risk score (GRS_CAD_) was calculated using 158 CAD-related genetic loci. Physical activity during the last four weeks was assessed through standardized interviews, resulting in measures of (1) engagement in physical exercise and (2) the total metabolic equivalents of general physical activity per week (METh/week). Linear regression models were used to analyze the associations of physical activity and genetic risk with log-transformed CAC, adjusting for confounders age, sex, and education.

**Results:**

Participants not engaging in physical exercise had a 1.336-fold (95%-CI: 1.171 to 1.533) higher CAC compared to those who exercised. No association was found between METh/week and CAC. The GRS_CAD_ was associated with a 1.206-fold (95%-CI: 1.130 to 1.287) higher CAC per standard deviation. Interaction analyses indicated that the genetic effect on CAC was slightly stronger in participants with higher METh/week levels, showing a 1.306-fold (95%-CI: 1.145 to 1.490) higher CAC per standard deviation of the GRS_CAD_ in the highest METh/week quartile compared to 1.109-fold (95%-CI: 0.978 to 1.259) higher CAC in the lowest quartile. No interaction was observed for engagement in physical exercise.

**Conclusions:**

While physical activity in sum was associated with lower levels of CAC, individuals reporting higher physical activity levels may also be less exposed to other non-genetic risk factors for CAD, leading to a slightly stronger association of genetic factors with CAC.

## Introduction

Coronary artery disease (CAD) stands as the leading cause of death globally representing a significant public health challenge [1–5]. Within the European Union, CAD is the second leading cause of death for individuals under the age of 65 accounting for approximately 17% of the overall mortality among women and 24% among men [6]. CAD is primarily caused by the buildup of calcification in the coronary arteries (CAC), which compromises blood flow and leads to a deficiency in oxygen and essential nutrients to the myocardium [7, 8]. This can result in a range of clinical manifestations, including myocardial infarction and cardiac death [9]. The development of CAD is influenced by a variety of risk factors [10–13], such as older age, male sex, high blood pressure, hyperlipidemia, smoking, and diabetes mellitus [14–16]. Protective lifestyle factors, particularly physical activity, also play a significant role in the development and progression of CAC [17, 18], subsequently lowering the risk of developing CAD [19–24].

In addition to these risk factors, genetic predisposition significantly contributes to the risk of CAD. The estimated heritability of CAD ranges between 40% and 60% [25]. Genome-wide association studies (GWAS) have identified numerous genetic variants robustly associated with an increased incidence of CAC and CAD [26]. However, the effect size of individual genetic variants on CAD phenotypes are generally low to moderate [27]. This modest impact of single genetic variants suggests the involvement of interactions between genetic risks and non-genetic risk factors such as low physical activity, which are increasingly recognized as important in the development of CAD [28].

Understanding the interactions between genetic risk and physical activity could provide valuable insights into the pathogenesis of CAD and inform personalized prevention strategies. Furthermore, understanding of how lifestyle modifications can mitigate genetic risk, potentially guiding public health policies and therapeutic approaches aimed at reducing the burden of CAD.

The aim of our study was to investigate whether a genetic risk score for CAD interacts with indicators of physical activity in a population-based cohort study to influence CAC levels.

## Methods

### Study population

Data of the Heinz Nixdorf Recall study, a population-based prospective cohort study, was used [29]. In this study, 4814 women and men, 45 to 74 years of age, with European ancestry participated. Individuals were randomly selected from a mandatory register of residents within the urban Rhein-Ruhr area in western Germany (cities Bochum, Essen, and Mülheim/Ruhr) between December 2000 to June 2003. The overall baseline response portion was 55.8% [30]. Informed consent was obtained from all participants. This study was approved by the ethics committee of the University Duisburg-Essen (25-12430-BO). Participants were involved in the design and conduct of our research.

### Coronary artery calcification and incident coronary events

To quantify CAC at the baseline examinations, a noncontrast-enhanced electron beam computed tomography using a C-150 scanner was used (GE Imatron, South San Francisco, CA). Details have been described elsewhere [29, 31]. The Agatston score was computed as a measure of total CAC defined as the sum of the area (in mm^2^) of each detectable focus in the epicardial coronary system multiplied by its computed tomography density, zero indicating a nondetectable calcification.

Using annual postal questionnaires including questions on current health status (i.e., medication, hospital admission, and outpatient diagnosis of coronary events), incident coronary events were assessed during follow-up. In case of a reported coronary event or a fatal event as much information as possible was obtained. Hospital records and records of the participants attending physician were then reviewed to verify each incident event. The endpoint for this study was unequivocally documented incident coronary death or nonfatal myocardial infarction which met the predefined study criteria [32]. Those criteria consisted of enzyme levels (levels of creatine kinase), troponin T or I, symptoms, electrocardiographic sign, as well as necropsy. An independent expert committee was appointed to validate endpoints over a median follow-up period of 15.8 (interquartile range of 11.03–18.8) years.

### Assessment of physical activity and covariates

Self-reported physical activity was assessed using standardized computer-assisted face-to-face interviews at study baseline according to the coding scheme of Ainsworth et al. ^33^. Participants were asked to list every type of physical exercise they practiced in the past four weeks as well as the frequency of exercise units and their duration (question: “*Please list*,* in order*,* all the sportive exercises you have participated in over the past 4 weeks. For each sport*,* please indicate how often and for how long you typically participate in it.*”). Furthermore, data on leisure time activities such as gardening or walking were collected. Each leisure time activity was enlisted with frequency and duration (question: “*Now let’s talk about other physical activities you do in your free time. These might include gardening or going for walks. Please list all the activities you’ve done over the past 4 weeks*,* one by one. For each activity*,* please indicate how often and for how long you usually do it.*”). For statistical analysis, two different measures were derived: (1) “no physical exercise” was defined as having reported no sportive exercise during the last 4 weeks with participants who reported ≥ 1 unit of exercise during the last 4 weeks as reference category; (2) the total number of metabolic equivalents in MET hours per week (i.e., “METh/week”) as a continuous measure of physical activity in a typical week of the participant was calculated combining information on frequency and duration of physical (i.e., sportive) exercise and leisure time activities during the past 4 weeks [33].

Standardized interviews at baseline examination were also used to collect information on potential confounders. Total years of formal education were coded combining information on school and vocational training according to the International Standard Classification of Education [34]. For stratified analyses, education was categorized into two groups. The lowest group being of ≤ 10 years (equivalent to a basic school degree with no vocational training), and the highest educational group of more than 10 years of education (equivalent to basic general education, leading to a vocational or university entrance qualification). Smoking status was dichotomized as current smoking (smoking cigarettes during the past year) versus former and never smoking. Alcohol intake was calculated as gram per week estimated from information on the total number of alcoholic drinks by type of drink (beer, wine, sparkling wine, and spirits). Based on a food frequency questionnaire, a dietary pattern index was calculated to determine the quality of the participants’ diet (i.e., the higher the dietary pattern index score, the healthier the diet).

### Genetic data

Blood samples were used for genotyping with different Illumina microarrays. Participants with missing genotype data > 5% and SNPs with minor an allele frequency of < 1%, a missing genotype frequency of > 5% or a deviation from Hardy-Weinberg equilibrium were excluded. SNPs used to calculate a weighted polygenic risk allele sum score (GRS_CAD_) were identified using the review by Erdmann et al. ^26^, which presents 163 CAD-related genetic risk loci with corresponding beta estimates for European populations detected and validated in several GWAS meta-analyses [26]. These beta estimates were used as weighting factors to calculate the GRS_CAD_. The present cohort was not part of the meta-analyses that derived the weighting factors, but was also of European descent. Information on 158 of the SNPs (in linkage disequilibrium of r^2^ < 0.2) was available in our dataset and was used to construct a weighted genetic risk allele sum score by (1) summing the number of risk alleles per participant times the corresponding SNP beta-estimate as a weighting factor, (2) then dividing the results by the sum of the SNP beta-estimates to rescale the GRS_CAD_ to the number of risk alleles. The GRS_CAD_ explained r^2^ = 0.3% of the CAC variance in the study population.

### Statistical analysis

Out of 4814 participants of the study, 3938 participants had non-missing information on genotypes, non-missing information on coronary artery calcification, as well as non-missing information on physical activity measures, and were free of CAD (i.e., history of myocardial infarction or coronary revascularization) at study baseline (Figure S1).

Linear regression models were fitted to calculate beta estimates and 95% confidence intervals (CI) for associations of physical activity and GRS_CAD_ with CAC adjusted for the confounders age, sex and socioeconomic position indicator education. The confounder selection to identify minimal adjustment sets was based on using direct acyclic graphs (DAG). A sensitivity analysis assuming lifestyle factors such as smoking, diet and alcohol intake as additional confounders was also conducted. There was no multicollinearity detected between variables included in the models. A log_e_ transformation of (CAC + 1) was applied to normalize the distributions of the CAC score and avoid numeric errors. Effect size estimates and 95% CIs were presented back transformed as exp(β) to interpret results on the original scale. Exp(β) can be interpreted as the relative change in the geometric mean of CAC per unit increase in the independent variable. The age- and sex-adjusted association between GRS_CAD_ and CAC was then assessed stratified by “no physical exercise” vs. “physical exercise” and additionally by METh/week quartiles. In addition, linear regression models adjusted for sex, age, and education were fitted including the main effects of GRS_CAD_ and the respective physical activity measure in addition to an interaction term between them. Main analyses were repeated using incident coronary events as outcome in Cox proportional hazard regression models. Validity of the proportional hazards assumption was confirmed prior to analysis and assessed by inspection of Schoenfeld residuals.

## Results

The study population of 3938 participants consisted of 2054 women (52.2%) and 1884 men (47.8%) with an average age of 59.4 years (Table 1). The median CAC score was 13.4 (interquartile range, 0-117.8) for the study population, with participants not reporting physical exercise having a higher median score than those engaged in physical exercise (19.3 (interquartile range, 0.0-130.0) versus 8.8 (interquartile range, 0.0-100.8)). In total, 291 incident coronary events occurred since study baseline, with a higher incidence reported for participants reporting no physical exercise. The overall incidence rate of coronary events during follow up was ~ 51 events per 10.000 person-years. Low educational attainment was more prevalent in participants reporting no physical exercise (9.6%) compared to men (5.1%). Participants had on average ~ 160 CAD-risk increasing alleles, with no difference between physical exercise groups. The difference in physical activity between groups was reflected in METh/week values with lower values in participants reporting no physical exercise. Current smoking was more prevalent in the group of reporting no physical exercise, while alcohol intake slightly lower in the physical exercise group. Quality of diet was slightly higher in the group reporting physical exercise.

**Table 1:** Characteristics of study participants (*n* = 3938) stratified by physical exercise

	all	no physical exercise	physical exercise
*n*	3938 (100%)	2007 (51.0%)	1931 (49.0%)
Female sex	2054 (52.2%)	1033 (51.5%)	1021 (52.9%)
age	59.4 (± 7.7)	59.6 (± 7.8)	59.1 (± 7.6)
Low education	445 (11.3%)	259 (12.9%)	186 (9.6%)
Current smoking	912 (23.2%)	522 (41.4%)	390 (22.1%)
Alcohol (g/week)	13.9 (0.0; 66.9)	7.4 (0.0; 60.0)	17.7 (0.0; 69.5)
Dietary pattern index	12.7 (± 3.1)	12.2 (± 3.1)	13.0 (± 3.1)
GRS_CAD_	160.4 (± 8.5)	160.2 (± 8.5)	160.4 (± 8.5)
METh/week	30.8 (14.0; 56.5)	21.0 (7.0; 44.5)	38.3 (21.8; 65.0)
Coronary events	291 (7.4%)	183 (9.1%)	108 (5.6%)
CAC (Agatston Score)	13.4 (0; 117.8)	19.3 (0; 130.0)	8.8 (0; 100.8)
Log_e_ (CAC + 1)	2.67 (0; 4.78)	3.01 (0; 4.88)	2.28 (0; 4.62)

Table 2 shows the results of the linear regression analysis to assess the association of no physical exercise, METh/week and the GRS_CAD_ with CAC. Those who were not engaged in physical exercise had on average a 1.336-fold higher CAC (95% CI, 1.171 to 1.533) compared to the group engaged in physical exercise. However, METh/week including all physical activities did not seem to be associated with CAC at baseline. Per standard deviation increase in the genetic risk score, CAC was on average 1.206-fold higher (95% CI, 1.130 to 1.287).

**Table 2:** Association of no physical exercise and METh/week (per 10 units increase) with coronary artery calcification (adjusted for age, sex, education) and of GRS_CAD_ (per standard deviation) with coronary artery calcification (adjusted for age, sex) from separate linear regression models (*n* = 3938)

	Exp(β )	95% CI	*P* value
No physical exercise	1.336	1.171 to 1.533	1.62x10^− 5^
METh/week (per 10 units)	1.004	0.990 to 1.019	0.560
GRS_CAD_ (per SD)	1.206	1.130 to 1.287	1.70x10^− 8^

**Fig. 1:**
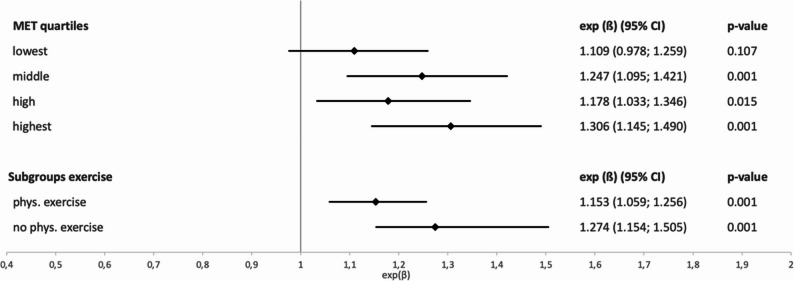
Age and sex adjusted effects of the GRS_CAD_ (per standard deviation) on coronary artery calcification (CAC) stratified by METh/week quartiles and physical exercise from separate linear regression models (*n* = 3938)

Figure 1 shows that the age and sex adjusted effect of the GRS_CAD_ on CAC was strongest in the highest METh/week quartile (exp(β) 1.306 (95% CI 1.145–1.490) per standard deviation) while in the lowest METh/week quartile the genetic effect was substantially less strong (exp(β) 1.109 (95% CI 0.978–1.259)). The subgroup of participants reporting no physical exercise showed a stronger association with CAC (Exp(β): 1.274, 95% CI, 1.154–1.505) than the subgroup with physical exercise (Exp(β ): 1.153, 95% CI: 1.059–1.256). This observation was also supported by estimated slopes for age and sex adjusted effects of the GRS_CAD_ on CAC stratified by METh/week quartiles and physical exercise (figure S2).

Table 3 shows the results of the linear regression model including a GRS_CAD_ by no physical exercise interaction term. Results gave no indication for an interaction of the GRS_CAD_ physical exercise. Results additionally including smoking, diet and alcohol intake as potential confounders led to comparable results (Table S4).

**Table 3:** Age, sex and education adjusted effects (exp(β)) of the GRS_CAD_ on CAC including an GRS_CAD_ by no physical exercise interaction term (*n* = 3938)

	Exp(β )	95% CI	*P* value
Constant	0.022	0.004 to 1.122	1.59*10^− 5^
Age (per year)	1.115	1.105 to 1.124	1.19x10^− 128^
Female Sex	0.167	0.147 to 0.191	6.381x10^− 143^
Low Education	1.051	0.850 to 1.301	0.644
No physical exercise	0.195	0.017 to 2.300	0.194
GRS_CAD_ (per standard deviation)	1.154	1.057 to 1.259	0.001
GRS_CAD_ x No physical exercise	1.107	1.133 to 1.261	0.125

Table 4 shows the results of the linear regression model including a GRS_CAD_ by METh/week interaction term. The exp(β) of 1.015 (95% CI: 1.0-1.03) observed for the interaction term gave indication for an interaction that shows stronger genetic effects on CAC with higher METh/week. In the model including a GRS_CAD_ by METh/week interaction term the METh/week main effect showed indication for negative association with CAC. Results additionally including smoking, diet and alcohol intake as potential confounders led to comparable results (Table S5).

**Table 4:** Age, sex and education adjusted effects (exp(β)) of the GRS_CAD_ on CAC including an GRS_CAD_ by METh/week interaction term (*n* = 3938)

	Exp(β )	95% CI	*P* value
Constant	0.034	0.006 to 0.203	2.21*10^− 4^
Age (per year)	1.115	1.105 to 1.125	7.3x10^− 126^
Female Sex	0.167	0.146 to 0.191	3.95x10^− 141^
Low Education	1.110	0.897 to 1.372	0.339
METh/week (per 10 units)	0.758	0.575 to 0.999	0.049
GRS_CAD_ (per standard deviation)	1.132	1.034 to 1.239	0.007
GRS_CAD_ x METh/week	1.015	1.000 to 1.030	0.045

Age and sex adjusted effects for no physical exercise in the last four weeks, METs per 10 units increase and the genetic risk score per SD on incident coronary events were derived from separate Cox proportional hazard regression models (Table S1). Results were directional consistent to those obtained for using CAC as outcome. Regression models including interaction terms showed no indication for an interaction between the GRS_CAD_ and both physical activity indicators (Tables S2 and S3).

## Discussion

The present study gave some indication for a weak positive interaction between the genetic risk for CAD and METh/week, reflecting levels of physical exercise and general physical activity on CAC in a population-based cohort. Stronger genetic effects on CAC were observed in groups of higher METh/week. No indication for interaction was present using a dichotomized measure of regular exercise as indicator for physical activity. While the interaction effect size measures were directionally consistent, no strong indication for an interaction was observed using incident coronary events as study outcome. While there were differences in socio-demographic and lifestyle factors between physical exercise groups, results of regression analysis were adjusted for these potential confounders.

Not engaging regularly in physical exercise was identified as being associated with higher CAC. These results were in line with previous studies [35]. METh/week were not associated with CAC in a regression model not accounting for the observed interaction with the CAD-related genetic risk score. In the model accounting for interaction, however, the estimate for the METh/week main effect indicated an association of METh/week with CAC that was directionally consistent with previous studies [36].

Stratification of the genetic effect by METh/week quartiles revealed a slightly stronger effect within higher levels of total physical activity. In contrast, a dichotomous measure of physical exercise showed no strong indication of modifying the genetic risk for CAD. This suggested that higher levels of general physical activity – comprising not only vigorous exercise but also moderate activities such as walking or cycling – may have a protective effect on adverse CAC levels directly, while at the same time modifying the genetic association with CAC in the opposite direction. One possible explanation for this observation could be that physical activity may help to mitigate other CAD risk factors such as metabolic syndrome or obesity. Individuals with higher physical activity levels may then experience stronger genetic effects as a result of lacking other non-genetic risk factors. Thus, the present results may indicate a statistical artefact, rather than evidence for biological interaction, and highlight the role of physical activity in reducing traditional cardiovascular risk factors, potentially uncovering the genetic predisposition to CAD in populations with healthier lifestyles.

Other studies have also explored the relationship between physical activity and a GRS for CAD [37]. Tikkanen and colleagues used the short International Physical Activity Questionnaire (IPAQ) to measure METs, which includes both vigorous and moderate activities. However, their study did not observe significant differences based on METs. This may be a consequence of selection in the study population, i.e., the UK Biobank, which predominantly included individuals from higher socio-economic status backgrounds with overall healthier lifestyle and lower disease risks [38].

In contrast to the observed weak interaction between the GRS_CAD_ and METh/week on CAC, no strong indication for such interaction was found for incident CAD events, while the interaction effect size measures were directionally consistent for both outcomes. This may reflect lower statistical power in analyzing a dichotomous outcome such as incident CAD events, but also adds to the fragile nature of the weak interaction observed for CAC. Compared to CAC as a marker for subclinical atherosclerosis, clinical CAD events reflecting additional etiological factors, such as acute inflammatory responses or plaque rupture [39]. Different results in investigating both outcomes may thus reflect different ways of how physical activity is being protective. While moderate physical activity may reduce the risk of developing atherosclerosis, it may not necessarily be protective via the same pathways in preventing more severe clinical events.

Strengths of the present study were its population-based design and long follow-up. However, a potential limitation was the cross-sectional design for the association analysis between physical activity and CAC. Thus, reverse causation cannot be ruled out, but as prevalent CAD cases were excluded from the analyses and subclinical CAC may have no strong impact on the level of physical activity, biased effect estimates as a result of reverse causation may be unlikely. Due to the moderate sample size the statistical power was limited for single SNP interaction analyses. However, by combining genetic information in a genetic risk score, statistical power was > 90% to detect the observed interaction assuming a two-sided alpha of 0.05 with the given sample size and the respective parameter distributions. There were additional limitations of this observational study that may have distorted the results, such as limited transferability of the results to populations of different ethnicity, CAC measure as a surrogate marker may not reflect atherosclerosis entirely, and potential residual confounding not accounted for. While the GRS used was robustly associated with CAC in the present study, it has not been clinically validated for CAC and the variance of CAC explained by the GRS was low. This adds to the cautious interpretation of the present results. In addition, the assessment of physical activity was based on self-reports, not on objective device-based measurement, and covered only a short recall window of four weeks. It also combined structured exercise with general leisure activity. This may have led to measurement error and biased results.

Under the assumption of valid study results, stronger genetic effects of a CAD-related genetic risk score on CAC were observed at higher levels of general physical activity. While physical activity in sum is associated with lower levels of CAC, findings of the present study suggest a stronger association of genetic factors with CAC in individuals reporting higher physical activity levels. This may not be interpreted as an indication for biological interaction, but rather as a result of generally lower rates of non-genetic CAD-risk factors associated with physical activity, leading to stronger CAD related-genetic effects.

## Data Availability

Due to data security reasons (i.e., data contain potentially participant identifying information), the Heinz Nixdorf Recall Study does not allow sharing data as a public use file. However, others can access the data used upon request, which is the same way the authors of the present paper obtained the data. Data requests can be addressed to the corresponding author at recall@uk-essen.de.
